# A phase 1b study of venetoclax and azacitidine combination in patients with relapsed or refractory myelodysplastic syndromes

**DOI:** 10.1002/ajh.26771

**Published:** 2022-11-10

**Authors:** Amer M. Zeidan, Uma Borate, Daniel A. Pollyea, Andrew M. Brunner, Fernando Roncolato, Jacqueline S. Garcia, Robin Filshie, Olatoyosi Odenike, Anne Marie Watson, Ravitharan Krishnadasan, Ashish Bajel, Kiran Naqvi, Jiuhong Zha, Wei‐Han Cheng, Ying Zhou, David Hoffman, Jason G. Harb, Jalaja Potluri, Guillermo Garcia‐Manero

**Affiliations:** ^1^ Section of Hematology, Department of Internal Medicine Yale University and Yale Cancer Center New Haven Connecticut USA; ^2^ Division of Hematology and Medical Oncology Knight Cancer Institute, Oregon Health and Science University Portland Oregon USA; ^3^ Division of Hematology, Department of Medicine University of Colorado Aurora Colorado USA; ^4^ Center for Leukemia Massachusetts General Hospital Boston Massachusetts USA; ^5^ Department of Hematology St George Hospital Sydney New South Wales Australia; ^6^ Department of Medicine Dana‐Farber Cancer Institute Boston Massachusetts USA; ^7^ Department of Hematology St Vincent's Hospital Melbourne Victoria Australia; ^8^ Section of Hematology/Oncology University of Chicago Medicine and Comprehensive Cancer Center Chicago Illinois USA; ^9^ Department of Hematology Liverpool Hospital Sydney New South Wales Australia; ^10^ Department of Hematology University of Arizona Cancer Center Tucson Arizona USA; ^11^ Department of Clinical Hematology Peter MacCallum Cancer Center and The Royal Melbourne Hospital Melbourne Victoria Australia; ^12^ Research and Development Genentech Inc South San Francisco California USA; ^13^ Research and Development AbbVie Inc North Chicago Illinois USA; ^14^ Department of Leukemia The University of Texas MD Anderson Cancer Center Houston Texas USA

## Abstract

Patients with relapsed/refractory (R/R) higher‐risk myelodysplastic syndromes (MDS) have a dismal median overall survival (OS) after failing hypomethylating agent (HMA) treatment. There is no standard of care for patients after HMA therapy failure; hence, there is a critical need for effective therapeutic strategies. Herein, we present the safety and efficacy of venetoclax + azacitidine in patients with R/R MDS. This phase 1b, open‐label, multicenter study enrolled patients ≥18 years. Patients were treated with escalating doses of oral venetoclax: 100, 200, or 400 mg daily for 14 days every 28‐day cycle. Azacitidine was administered on Days 1–7 every cycle at 75 mg/m^2^/day intravenously/subcutaneously. Responses were assessed per modified 2006 International Working Group (IWG) criteria. Forty‐four patients (male 86%, median age 74 years) received venetoclax + azacitidine treatment. Median follow‐up was 21.2 months. Hematological adverse events of Grade ≥ 3 included febrile neutropenia (34%), thrombocytopenia (32%), neutropenia (27%), and anemia (18%). Pneumonia (23%) was the most common Grade ≥ 3 infection. Marrow responses were seen including complete remission (CR, *n* = 3, 7%) and marrow CR (mCR, *n* = 14, 32%); 36% (16/44) achieved transfusion independence (TI) for RBCs and/or platelets, and 43% (6/14) with mCR achieved hematological improvement (HI). The median time to CR/mCR was 1.2 months, and the median duration of response for CR + mCR was 8.6 months. Median OS was 12.6 months. Venetoclax + azacitidine shows activity in patients with R/R MDS following prior HMA therapy failure and provides clinically meaningful benefits, including HI and TI, and encouraging OS.

## 1 INTRODUCTION

Myelodysplastic syndromes (MDS) consist of a heterogeneous group of clonal hematopoietic stem cell disorders with significant morbidity and mortality, arising at a median age of diagnosis of 76 years.^1^ Hypomethylating agents (HMAs), azacitidine and decitabine, are the standard of care for patients with newly diagnosed higher‐risk MDS. Azacitidine improves survival compared to conventional treatments and was approved based on the AZA001 trial showing a median overall survival (OS) of 24.5 months versus 15.0 months, respectively.^2^ A recent trial in patients with higher‐risk MDS reported that those treated with azacitidine monotherapy had a median OS of 17.5 months.^3^ Objective responses were seen in about half of the patients treated with HMAs, although complete responses (CRs) in prior studies were observed in 10%–32% of patients.^3^, ^4^, ^5^, ^6^ Among patients with initial responses to HMAs, the median duration of response (DoR) is approximately 9–15 months.^2^ The prognosis of patients after azacitidine or decitabine failure is poor, with a dismal median OS of <6 months,^4^, ^6^, ^7^ and less than a third of patients survive to 1 year.^4^, ^6^, ^8^ Patients with relapsed/refractory (R/R) MDS have a high unmet medical need, with no existing standard of care after HMA therapy failure when an allogeneic hematopoietic stem cell transplant is not possible.

Several hematologic malignancies, including higher‐risk MDS, depend on the anti‐apoptotic protein B‐cell lymphoma‐2 (BCL‐2). Over‐expression of BCL‐2 is associated with tumor initiation, disease progression, and drug resistance and represents a potential therapeutic target.^9^ Venetoclax is a potent, selective oral inhibitor of BCL‐2 that blocks the activity of the pro‐survival BCL‐2 protein, priming the neoplastic cells for apoptosis.^10^, ^11^ Following HMA treatment, an increase in BCL‐2 and a decrease in MCL‐1 levels have been described; venetoclax treatment may reduce the apoptotic threshold in MDS and allow response to HMAs even in cells previously resistant to HMA treatment.^12^

In this phase‐1b study, we evaluated the safety of venetoclax either alone or in combination with azacitidine therapy after HMA failure. Due to the limited efficacy with venetoclax monotherapy, this report primarily focuses on the results of the combination of venetoclax and azacitidine in patients with R/R MDS.

## 2 METHODS

### 2.1 Study design

This open‐label, multicenter study in patients with R/R MDS consisted of three cohorts: Cohort‐1 evaluated venetoclax monotherapy at 400 mg once daily (QD) and 800 mg QD; Cohort‐2 included escalating doses of venetoclax (100, 200, and 400 mg) in combination with azacitidine, evaluated the dose‐limiting toxicities (DLTs), and determined the maximal tolerated dose (MTD); Cohort‐3 evaluated the safety and efficacy of venetoclax (400 mg QD) with azacitidine (Figure S1).

Patients ≥18 years of age with R/R MDS were enrolled. Patients enrolled in venetoclax monotherapy had documented failure of prior therapy with an HMA (HMA failure defined as relapse after initial complete remission [CR] or partial remission [PR] or hematologic improvement [HI] or failure to achieve any of these responses after at least four cycles of either azacitidine or decitabine within the last 5 years). Eligible patients had <20% bone marrow (BM) blasts per BM biopsy/aspirate at screening, not current candidates for allogeneic hematopoietic stem cell transplantation and had an Eastern Cooperative Oncology Group (ECOG) performance score of ≤2. Patients were excluded if they had myelodysplastic/myeloproliferative neoplasms or therapy‐related MDS, prior therapy with a BH3 mimetic, or had undergone prior transplantation. The local ethics committee approval was obtained, and the patients gave written informed consent. The study was conducted in accordance with the International Conference on Harmonization, Good Clinical Practice Guidelines, and the Declaration of Helsinki.

Venetoclax was administered orally, once daily, with food. Patients on venetoclax monotherapy received 400 or 800 mg once daily for a 28‐day cycle. Escalating doses of venetoclax were evaluated in combination with azacitidine for DLTs. Patients on the combination received venetoclax (100, 200, or 400 mg) during Days 1–14 of the 28‐day cycle. Azacitidine was administered at a standard dose of 75 mg/m^2^ on Days 1–7 every cycle. Treatment continued until the patient continued to benefit, or until the occurrence of unacceptable toxicity by investigator's discretion, withdrawal of consent, or if clinically indicated. During dose‐escalation, all patients received antibiotic prophylaxis for the first two cycles. Thereafter, antibiotic, antifungal prophylaxis, and granulocyte colony‐stimulating factors were used at the investigator's discretion.

### 2.2 Outcomes

Patients were monitored for safety throughout the study. The adverse events (AEs) were graded according to the National Cancer Institute Common Terminology Criteria for Adverse Events Version 4.03.^13^ Treatment‐emergent AEs (TEAEs), including clinical tumor lysis syndrome (TLS), were defined as those between the first dose and 30 days after the last dose of study treatment. Laboratory TLS was defined as reported by Howard et al.^14^ DLT was determined based on the AEs occurring during the DLT observation period defined as the first treatment cycle (Supporting Information). Recommendations for venetoclax dose adjustments were made due to hematologic toxicities (Supporting Information).

BM assessments were performed at the end of cycles 1, 2, 4, and 6, then every three cycles thereafter. Responses were assessed by the investigator per modified 2006 IWG criteria for MDS.^15^ Patients were considered evaluable for response if they had disease assessment before discontinuation of the study or study treatment. HI rate was assessed by improvement in either erythroid, platelet, or neutrophil counts for eligible patients. Red blood cell (RBC) or platelet transfusion independence (TI) was defined as no RBC or platelet transfusions in at least 56 days during the evaluation period, and the duration of post‐baseline TI was defined as the maximal time that a patient received no transfusion for at least 56 days during the evaluation period. Time to acute myeloid leukemia (AML) transformation was assessed and defined by the number of days from the date of the first dose of the study drug to the date of documented AML progression (blast count ≥ 20% in peripheral blood or BM).^15^

Blood samples were collected for venetoclax pharmacokinetics (PKs) analysis at 6 h post‐dose on Day 1 of cycle 1, pre‐dose and 2, 4, 6, 8, and 24 h post‐dose on Day 4 of cycle 2; and pre‐dose on Day 4 of cycles 3, 4, 6, and 8. Venetoclax concentrations from cycle 2, Day 4 were used to determine the *C*_max_, the time to *C*_max_ (*T*_max_), and the area under the plasma concentration‐time curve from time 0 to 24 h (AUC_24_), using non‐compartmental methods.

At baseline, mutation analyses were performed with mononuclear cells (i.e., BM *n* = 38; peripheral blood *n* = 3) from 41/44 (93%) patients with VariantPlex Myeloid/Core Myeloid NGS panel (Invitae) and TruSight Myeloid panel (Illumina, Inc.). The limit of detection for these panels was 1%–5%. The %BCL‐2+/%BCL‐xL blast ratio in 30/44 (68%) patients at baseline was determined as described previously by Konopleva et al.^16^

Patient‐reported outcomes (PROs) were assessed by Fatigue (PRO Measurement Information System [PROMIS] Fatigue Short Form [SF] 7a), Physical Function (PROMIS Physical Function SF 10a), Quality of Life (European Organization for Research and Treatment of Cancer Quality of Life Questionnaire Core 30 [EORTC‐QLQ‐C30]) and EuroQoL EQ‐5D‐5L. Data were collected at baseline and then on Day 1 of every other treatment cycle through cycle 15.

### 2.3 Statistical analyses

A Bayesian optimal interval design guided dose‐escalation. The safety and efficacy analyses were performed on patients who received at least one dose of venetoclax. Demographics were analyzed using descriptive statistics. DoR, OS, EFS, and TTNT were analyzed using the Kaplan–Meier methodology. Median estimates and corresponding 95% confidence intervals (CIs) were calculated.

For PK, plasma concentrations of venetoclax and azacitidine were calculated for each cohort and dose level, and summary statistics were computed for each parameter.

PROs were analyzed by cycle for proportions of patients whose change in scores (for EORTC‐QLQ‐C30 scales and PROMIS F) indicated meaningful improvement, deterioration, or stability. Differences in proportions were analyzed using the chi‐square test. Due to small sample sizes, data reported here are restricted to the first three post‐baseline cycles (cycles 3, 5, and 7). For the EORTC‐QLQ‐C30 scales, a threshold of five points was used, based upon the lower limit of meaningful change in scores.^17^ An increase of at least 5 points indicated improvement (better) in functioning; a decrease of at least 5 points indicated deterioration (worsening) of symptoms; stable patients were defined as those with score changes of less than 5 in either direction. The threshold for meaningful change in PROMIS F scores was a shift of at least 5 points, based upon the upper limit of the MID value range reported by Yost et al.^18^ Higher scores on the PROMIS F indicated more fatigue; thus, an increase of at least five points indicated meaningful deterioration while a decrease is indicative of meaningful improvement. Meaningful change in the EQ‐5D‐5L VAS for overall health status, based upon published estimates, was defined as a change of seven points on a 0–100 scale where higher scores indicated better health status.^19^ Meaningful improvement in health status was defined as a score increase of at least seven points and meaningful deterioration as a score decrease of at least seven points, with changes less than seven points indicating a stable health state.

## 3 RESULTS

### 3.1 Patients

At data cutoff date of April 30, 2021, 44 patients were enrolled in the venetoclax and azacitidine cohorts (venetoclax 100 mg [*n* = 10]; venetoclax 200 mg [*n* = 7], and venetoclax 400 mg including dose‐escalation and safety‐expansion [*n* = 27]). An additional 26 patients were enrolled in the venetoclax monotherapy cohorts (venetoclax 400 mg [*n* = 15] and venetoclax 800 mg [*n* = 11]); Due to limited efficacy, the venetoclax monotherapy regimen was discontinued. The median follow‐up of the study was 21·2 months (range, 0·4–37·5).

The baseline and clinical characteristics of patients who received venetoclax and azacitidine are summarized in Table 1, and those who received venetoclax monotherapy are shown in Table S1. The median age of patients treated with venetoclax and azacitidine was 74 years (range, 44–91), and 38 (86%) were male. The median number of prior HMA therapies was 1 (range, 1–2), and 28 (65%) patients received ≥6 cycles of therapy. The median BM blast count was 7·3% (range, 0–16). At study entry, the International Prognostic Scoring System‐Revised (IPSS‐R) scores were low, 9%; intermediate, 18%; high, 36%; and very high, 36%. The most frequently mutated genes identified were *ASXL1* (39%), *RUNX1* (25%), and *DNMT3A* (16%).

**TABLE 1:** Baseline and clinical characteristics of patients treated with venetoclax and azacitidine

Characteristic	Venetoclax + azacitidine (*N* = 44)
Sex, *n* (%)	
Male	38 (86·3)
Female	6 (13·7)
Race, *n* (%)	
White	43 (97·7)
Black or African American	1 (2·3)
Age, years, median (range)	74 (44–91)
ECOG performance score, *n* (%)	
0	10 (22·7)
1	27 (61·3)
2	7 (15·9)
Bone marrow blasts, *n* (%)	
<5%	11 (25·0)
≥5%–20%	33 (75·0)
Median bone marrow blast count, % (range)	7·3 (0–16·0)
Number of prior therapies, median (range)	1 (1–2)
Number of prior HMA therapies, *n* (%)	
1	42 (97·7)
2	1 (2·3)
Missing	1
Type of prior HMA therapy, *n* (%)	
Azacitidine	31 (70∙5)
Decitabine	13 (29∙5)
Guadecitabine	1 (2∙3)
Number of prior HMA cycles completed at baseline, *n* (%)	
≤6 cycles	15 (34·9)
>6 cycles	28 (65·1)
Missing	1
HMA failure, *n* (%)	
Primary	32 (74·4)
Other^a^	11 (25·6)
Missing	1
IPSS cytogenetic risk groups, *n* (%)	
Good	24 (54·5)
Intermediate	13 (29·5)
Poor	7 (15·9)
IPSS‐R risk categories, *n* (%)	
Low	4 (9·1)
Intermediate	8 (18·1)
High	16 (36·4)
Very high	16 (36·4)
Genetic mutations,^b^ *n* (%)	
*TP53*	5 (11·3)
*RUNX1*	11 (25·0)
*TET2*	6 (13·6)
*ASXL1*	17 (38·6)
*SRSF2*	6 (13·6)
*IDH2*	6 (13.6)
*SF3B1*	4 (9·1)
*DNMT3A*	7 (15·9)
*EZH2*	5 (11·4)
*BCORL1*	1 (2·3)
Not detected/missing/not evaluable	3 (6·8)

Abbreviations: ECOG, Eastern Cooperative Oncology Group; HMA, hypomethylating agent; IPSS‐R, International Prognostic Scoring System—Revised; mCR, complete remission with incomplete marrow remission; RBC, red blood cell.

^a^ Other: Either primary or secondary HMA failure.

^b^ Mutations assessed from bone marrow and peripheral blasts.

### 3.2 Safety

Patients received a median of four cycles (range, 1–32) of venetoclax and four cycles (range, 1–31) of azacitidine. All patients treated with venetoclax and azacitidine experienced at least one AE (Table 2 and Table S2). The most common Grade ≥ 3 hematologic AEs experienced by ≥15% of patients included febrile neutropenia (34%), thrombocytopenia (32%), neutropenia (27%), and anemia (18%). Commonly occurring Grade ≥ 3 non‐hematologic AEs experienced by ≥25% of patients were nausea (48%), constipation (46%), diarrhea (39%), and fatigue (36%). Pneumonia (23%) was the most common Grade ≥ 3 infection. Serious AEs ≥10% occurrences were febrile neutropenia (23%) and pneumonia (18%). No DLTs were observed. The MTD was not reached in any cohort. Venetoclax dose of 400 mg per day for 14 days was recommended for the safety‐expansion cohort based on the safety in combination with 75 mg/m^2^ azacitidine per 28‐day cycle. No dose‐escalation beyond 400 mg in combination with azacitidine was performed. The patients who received the recommended phase 2 dose (RP2D) of venetoclax 400 mg per 14 days cycle in combination with azacitidine experienced AEs at rates comparable to those at all dose levels of venetoclax. All AEs at the RP2D of 400 mg were manageable with standard supportive care. Four (9%) deaths occurred within ≤30 days after the last dose of study treatment (pneumonia [*n* = 1]; considered possibly related to both venetoclax and azacitidine treatment, sepsis [*n* = 1], septic shock [*n* = 1], and gastrointestinal hemorrhage [*n* = 1]). Overall, 33 (75%) patients receiving combination treatment discontinued the study. The primary causes of study discontinuation were death 29 (66%), patient withdrawal 3 (7%), and lost‐to‐follow‐up 1 (2%). Nine (21%) patients discontinued venetoclax due to TEAE, of which only 1 (2%) was due to thrombocytopenia. Five (11%) patients had a reduction in venetoclax dose duration due to AEs, which included neutropenia (5%), cardiac disorders (2%), fatigue (2%), and renal impairment (2%). Twenty‐one (48%) patients experienced AEs that led to venetoclax interruption primarily due to febrile neutropenia (16%), neutropenia (9%), and pneumonia (7%). Twenty (46%) patients had >2 venetoclax dose interruptions. The AEs experienced by patients treated with venetoclax monotherapy (400 and 800 mg) are also summarized in Table 2.

**TABLE 2:** Safety profile of patients on venetoclax and azacitidine treatment

	Venetoclax 100 mg + azacitidine 75 mg/m^2^	Venetoclax 200 mg + azacitidine 75 mg/m^2^	Venetoclax 400 mg + azacitidine 75 mg/m^2^	All venetoclax 400 mg + azacitidine 75 mg/m^2^	Venetoclax monotherapy (400 or 800 mg)	Total
	(*N* = 10)	(*N* = 7)	(*N* = 27)	(*N* = 44)	(*N* = 26)	(*N* = 70)
	*n* (%)	*n* (%)	*n* (%)	*n* (%)	*n* (%)	*n* (%)
Any adverse event	10 (100·0)	7 (100·0)	27 (100·0)	44 (100·0)	26 (100·0)	70 (100·0)
Hematologic						
Febrile neutropenia	3 (30·0)	4 (57·1)	8 (29·6)	15 (34·1)	6 (23·1)	21 (30·0)
Thrombocytopenia	4 (40·0)	1 (14·3)	9 (33·3)	14 (31·8)	4 (15·4)	18 (25·7)
Neutropenia	4 (40·0)	2 (28·6)	6 (22·2)	12 (27·3)	5 (19·2)	17 (24·3)
Anemia	2 (20·0)	0	6 (22·2)	8 (18·2)	4 (15·4)	12 (17·1)
Non‐hematologic						
Nausea	4 (40·0)	5 (71·4)	12 (44·4)	21 (47·7)	10 (38·5)	31 (44·3)
Constipation	6 (60·0)	3 (42·9)	11 (40·7)	20 (45·5)	4 (15·4)	24 (34·3)
Diarrhea	6 (60·0)	1 (14·3)	10 (37·0)	17 (38·6)	9 (34·6)	26 (37·1)
Fatigue	3 (30·0)	1 (14·3)	12 (44·4)	16 (36·4)	7 (26·9)	23 (32·9)
Pneumonia	2 (20·0)	1 (14·3)	7 (25·9)	10 (2·7)	4 (15·4)	14 (20·0)
Decreased appetite	3 (30·0)	3 (42·9)	5 (18·5)	11 (25·0)	3 (11·5)	14 (20·0)
Dyspnea	3 (30·0)	1 (14·3)	5 (18·5)	9 (20·5)	5 (19·2)	14 (20·0)
Any ≥ grade 3 adverse event	10 (100·0)	7 (100·0)	25 (92·6)	42 (95·5)	22 (84·6)	64 (91·4)
Febrile neutropenia	3 (30·0)	4 (57·1)	8 (29·6)	15 (34·1)	6 (23·1)	21 (30·0)
Thrombocytopenia	4 (40·0)	1 (14·3)	9 (33·3)	14 (31·8)	4 (15·4)	18 (25·7)
Neutropenia	4 (40·0)	2 (28·6)	6 (22·2)	12 (27·3)	5 (19·2)	17 (24·3)
Anemia	2 (20·0)	0	6 (22·2)	8 (18·2)	4 (15·4)	12 (17·1)
Pneumonia	2 (20·0)	1 (14·3)	7 (25·9)	10 (22·7)	4 (15·4)	14 (20·0)
Any serious AEs	7 (70·0)	4 (57·1)	16 (59·3)	27 (61.4)	20 (76·9)	47 (67·1)
Febrile neutropenia	3 (30·0)	3 (42·9)	4 (14·8)	10 (22·7)	3 (11·5)	13 (18·6)
Pneumonia	2 (20·0)	1 (14·3)	5 (18·5)	8 (18·2)	4 (15·4)	12 (17·1)
Any TEAE with a reasonable possibility of being related to venetoclax	9 (90·0)	6 (85·7)	26 (96·3)	41 (93·2)	22 (84·6)	63 (90·0)
Any TEAE with a reasonable possibility of related to azacitidine	10 (100·0)	6 (85·7)	27 (100·0)	43 (97·7)	0	43 (61·4)

*Note*: Listed AEs include ≥20% in all treated patients; Grade ≥3 or higher AEs include ≥15% in all treated patients; Serious AEs in ≥10% in all treated patients.

### 3.3 Efficacy

**FIGURE 1:**
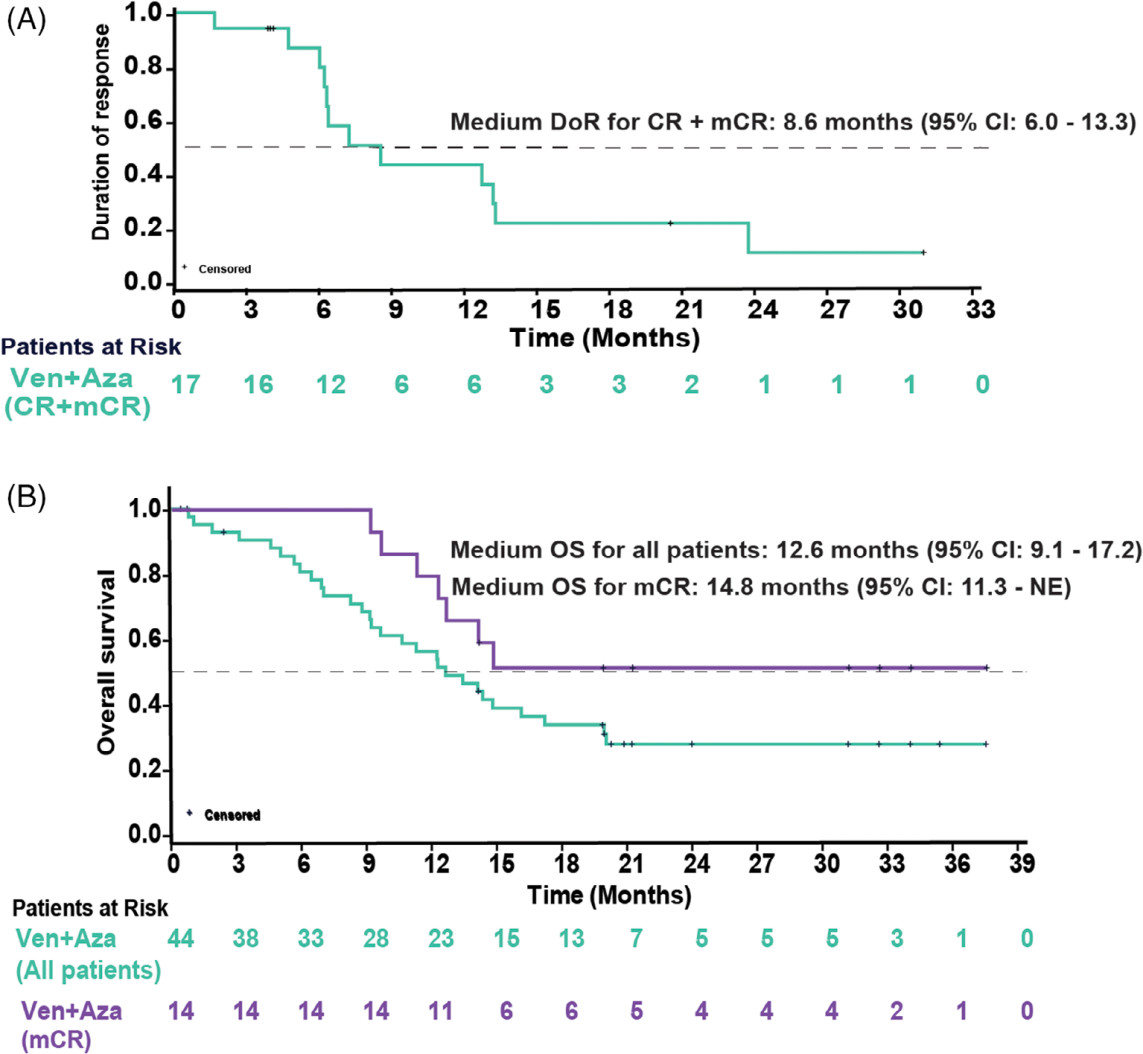
(A) Duration of response (DoR). (B) Overall survival (OS) in patients treated with venetoclax (Ven) and azacitidine (Aza). [Color figure can be viewed at wileyonlinelibrary.com]

Among 37 patients evaluable for a response, mORR was observed in 17 (39%), of which CR and marrow CR (mCR) was achieved by 3 (7%) and 14 (32%) patients, respectively (Table 3). Non‐responders included stable disease in 18 (41%) patients, and 2 (5%) had progressive disease. The median time to first response for mORR was 1·2 months (range, 0·7–6·3). The median DoR for mCR was 8·6 months (95% CI, 6·0–23·8; Figure S2) and mORR was 8·6 months (95% CI, 6·0–13·3; Figure 1A). 6/14 (43%) patients who achieved mCR also achieved HI. Additionally, 36% (16/44) achieved post‐baseline transfusion independence (TI) for both RBC and platelet, and the median duration of RBC and platelet TI was 4.3 months (range, 2·3–17·8). Nine (21%) progressed to AML with median time to AML progression of 4.9 months (range, 0·00–19·80). The median OS for all patients (*n* = 44) was 12.6 months (95% CI, 9·1–17·2). The median OS for patients with mCR (*n* = 14) was 14·8 months (95% CI, 11·3–NE; Figure 1B). The median TTNT was 5·7 months (95% CI, 4·8–8·8). The median PFS was 8·6 months (95% CI, 5·4–14·3), and the median event‐free survival was 6·9 months (95% CI, 5·1–8·8). Nine (21%) patients received a post‐study transplant. Four patients who underwent transplant died by the time of data cutoff; the duration of OS ranged between 8·3 and 37·5 months. The median OS censoring transplant was 12.2 months (95% CI, 8.8–14.3). The efficacy of the venetoclax monotherapy regimen was limited (one mCR, one patient with TI for both RBC and platelet; Table S3).

**TABLE 3:** Efficacy among patients treated with venetoclax and azacitidine

	Venetoclax + azacitidine (*N* = 44)
Duration of study follow‐up, months, median (range)	21·2 (0·4–37·5)
Response rates, *n* (%)	
mORR	17 (38·6)
CR	3 (6·8)
mCR	14 (31·8)
Not evaluable	7 (15·9)
Time to response, months, median (range)	
Time to mORR	1·2 (0·7–6·3)
Time to mCR	1·4 (0·7–6·3)
Duration of response (DoR), months, median (95% CI)	
DoR for mORR	8·6 (6·0–13·3)
DoR for CR	9·1 (6·3–NE)
DoR for mCR	8·6 (6·0–23·8)
Composite response rate^a^ (CR + PR + mCR + HI^b^), *n*/*N* (%)	19/44 (43·2)
Composite response of mCR + HI, *n* (%)	6/14 (42·9)
Post‐baseline transfusion independence (TI) rate, *n* (%)	
RBC	18 (40·9)
Platelet	24 (54·5)
RBC and platelet	16 (36·4)
Maximum duration of post‐baseline TI, months, median (range)	
RBC	4·0 (2·3–20·0)
Platelet	4·8 (2·0–25·6)
RBC and platelet	4·3 (2·3–17·8)
Post‐baseline TI^c^ rate for patients who were transfusion dependent at baseline, *n*/*N* (%)	
RBC transfusion dependent at baseline	11/32 (34·4)
Platelet transfusion dependent at baseline	7/15 (46·7)
RBC or platelet transfusion dependent at baseline	10/32 (31·3)
Maximum duration of post‐baseline TI for patients who were transfusion dependent at baseline, months (range)	
RBC	4·0 (2·3–20·0)
Platelet	3·8 (2·0–7·9)
RBC and platelet	3·8 (2·3–7·8)
Transformation from MDS to AML, *n* (%)	9 (20·5)
Time to AML transformation, months, median (range)	4·9 (0·0–19·8)
Time to next treatment, months, median (95% CI)	5·7 (4·8–8·8)
Overall survival (OS), months, median (95% CI)	
OS	12·6 (9·1–17·2)
OS for mCR	14·8 (11·3–NE)
Progression‐free survival, months, median (95% CI)	8·6 (5·4–14·3)
Event‐free survival, months, median (95% CI)	6·9 (5·1–8·8)

*Note*: mORR = CR + PR + mCR.

Abbreviations: CR, complete remission; HI, hematologic improvement; mCR, marrow CR; mORR, Modified overall response rate; NE, not evaluable; PR, partial remission.

^a^ Patients who achieved any component of (CR + PR + mCR + HI).

^b^ HI = HI‐E + HI‐P + HI‐N; HI‐E, Transfusion dependent on packed red blood cells or whole blood 8 weeks prior to cycle 1 day 1 (C1D1) or hemoglobin level < 11 g/dl; HI‐P, Transfusion dependent on platelet 8 weeks prior to C1D1 or platelet counts <100 × 10^9^/L; HI‐N, Neutrophil counts <1.0 × 10^9^/L at baseline.

^c^ The post‐baseline transfusion independence rate is defined as a period of at least 56 days with no transfusion during the evaluation period. The evaluation period for transfusion independence is from the date of the first dose of the study drug to the last dose of the study drug +30 days or 1 day before the date of progressive disease from disease response (2006 IWG) electronic clinical report form, death, or the initiation of post‐treatment therapy whichever is earliest.

Patients with *IDH2* mutations (*n* = 6) achieved an ORR of 83%, whereas those with *TP53* mutations (*n* = 5) achieved a response of 20% (Table S4). Responses were observed in patients with other high‐risk mutations including *RUNX1* (*n* = 11; ORR 54%), *TET2* (*n* = 6; ORR 33%), and *DNMT3A* (*n* = 7, ORR 57%). Best responses and median OS were independent of baseline IPSS‐R risk categories. A heat map showing the relationship between IPSS‐R scores at the study baseline, response categories, and genetic mutations is shown in Figure S3, and median OS by IPSS‐R risk categories is shown in Supporting Information. Venetoclax and azacitidine also demonstrated clinical activity independent of %BCL‐2+/%BCL‐xL blast ratio (Figure S4).

### 3.4 Pharmacokinetics

Peak venetoclax concentrations were reached at a median of 6 h post‐dose. Venetoclax exposures (dose‐normalized maximum observed plasma concentration [*C*_max_] and area under the plasma concentration‐time curve from time 0 to 24 h [AUC_24_]) from venetoclax as monotherapy and in combination with azacitidine were generally comparable, supporting that coadministration with azacitidine does not affect the PKs of venetoclax (Table S5).

### 3.5 Patient‐reported outcomes

For the EORTC‐QLQ‐C30 scales, chi‐square tests of differences in proportions of responders revealed that most patients remained stable across most symptoms and QoL scores over the first three cycles. Significant differences in proportions of patients with improved, deteriorated, or stable scores were observed for several symptom scales for at least one cycle, including constipation, diarrhea, dyspnea, nausea and vomiting, and insomnia. Similarly, most patients remained stable with respect to their cognitive functioning and reported no change in financial difficulties during one or more cycles. For PROMIS F, no significant differences were found in the proportion of patients by responder type (improved, deteriorated, and stable). Trends showed most patients remained stable across all three cycles. Contrary to trends observed for the EORTC‐QLQ‐C30 and PROMIS F scores, overall health status measured by the EQ‐5D, worsened for a larger proportion of patients, but this was not consistent across all cycles. In summary, physical functioning, key symptoms, and overall health status scores from the EORTC‐QLQ‐C30 and PROMIS F remained stable for most patients across the first three post‐baseline cycles. Similarly, for cognitive function and symptom scores of the EORTC‐QLQ‐C30, most patients remained stable during one or more cycles.

## 4 DISCUSSION

Treatment of patients with MDS with a prior HMA failure remains a significant area of unmet clinical need. Patients have an adverse prognosis and limited treatment options. In this phase 1b study, venetoclax was administered both as monotherapy and in combination with azacitidine in patients with R/R MDS after HMA therapy, with several patients achieving meaningful clinical benefits with the combination. Alternately, the efficacy of venetoclax monotherapy was limited.

The patients treated in our study reflect the older patient population with R/R MDS with a median age of 74 years. The majority had very high or high‐risk IPSS‐R disease (73%). They were also heavily pretreated with HMAs, with a median of nine cycles of prior HMA therapy, and 65% received at least six cycles of prior HMA therapy.

The severity, incidence, and types of AEs observed with the combination of venetoclax and azacitidine were consistent with the known safety profile of both agents, and no new toxicities for venetoclax were identified. There was no occurrence of TLS, patients were treated in an outpatient setting, and there was no dose ramp‐up for venetoclax. The hematologic toxicities reported were predominantly neutropenia and thrombocytopenia. These known side effects of venetoclax treatment were noted in prior studies of venetoclax in AML.^20^, ^21^, ^22^ The AEs were managed by following standard medical practice guidelines. No DLTs were reported in the dose‐escalation cohorts, and the 30‐day mortality was below 5%.

The study demonstrated marrow responses including 7% CR (3/44) and 32% mCR (14/44). While the value of mCR as a response in MDS patients is debated, 6 of the 14 patients who achieved mCR also achieved HI, which is clinically meaningful and has been consistently associated with OS benefits in prior analyses.^23^ Further, a total of nine patients who achieved less than 5% blasts were able to proceed to allogeneic BM transplant, which is often an important goal in managing R/R MDS as allogeneic transplant is potentially the only curative therapy.

Notably, the responses were attained early within a median of 1.2 months of treatment and sustained for a median of 8·6 months; with a median OS of 12·6 months. The historic median OS for R/R MDS observed in prior studies is between 4·3 and 5·6 months.^6^, ^7^ While the median OS in our study appears to be promising, it must be noted that selection bias of patients enrolled in the study might have influenced the achieved OS with the combination in this single‐arm study, and only a randomized study would confirm if OS can be improved with this combination. Further, the contrast between limited responses with venetoclax monotherapy and the favorable responses seen in this heavily pretreated HMA population with the venetoclax add‐on strategy to HMA is intriguing and might suggest that venetoclax may synergize or re‐sensitize MDS cells to HMA therapy. Indeed, a recent small single‐center study that added venetoclax to HMA therapy among MDS patients with prior HMA failure also reported achievement of mCR in all six patients treated.^24^ Transfusion dependence is a major problem in R/R MDS patients. Patients often spend several days a week receiving blood and platelet transfusions in clinics, potentially impairing their quality of life and exposing them to potential complications of transfusions. In our study, 43% of patients achieved HI, and 36% achieved TI for both RBC and platelet, which are essential patient‐centered clinical benefits.

Further, the results of our study have been similarly replicated in other studies. A recent retrospective analysis of patients with R/R MDS treated with the combination reported an overall response rate of 44% and median OS of 11·4 months (95% CI, 5·7–not estimable).^25^ In contrast to the combination, the clinical activity among patients treated with venetoclax monotherapy was limited. One patient attained mCR, and the median OS was 6·7 months (95% CI, 3·8–19·7). These findings suggest that venetoclax therapy synergies and potentially re‐sensitizes MDS cells to HMA therapy.

We observed responses in several genetically mutated subgroups. *ASXL1*, *RUNX1*, and *DNMT3A* mutations are common in MDS.^26^ These mutations did not appear to differentially impact patient survival in this study, although interpretation is limited by the small number of patients in these subgroups. Among the less frequent mutations, patients harboring *TP53* mutations attained lower response rates and shorter OS, consistent with findings from other studies.^27^, ^28^ In patients with *IDH2* mutation, the response rate of 83% and median OS of 17·1 months are of interest and should be evaluated in future studies.

Our study should be interpreted with caution as it lacks a comparator arm. The small sample size of the dose‐escalation and safety‐expansion cohorts precluded a dose–response analysis of this combination in this R/R MDS population. Similarly, the small number of overall assessed cycles limited the interpretability of PROs.

The study demonstrated a manageable safety profile with meaningful clinical benefits with the combination of venetoclax and azacitidine in adult patients with R/R MDS. Additional data, potentially including other combinations with venetoclax should be explored in future studies to enhance clinical responses in this hard‐to‐treat patient population.

## AUTHOR CONTRIBUTIONS

*Conception and design*: All authors. *Development of methodology*: All authors. *Acquisition of data (provided animals, acquired and managed patients, provided facilities, etc.)*: All authors. *Analysis and interpretation of data (e.g., statistical analysis, biostatistics, computational analysis)*: All authors. *Writing, review, and/or revision of the manuscript*: All authors. All authors had access to relevant data and participated in the drafting, review, and approval of this manuscript. No honoraria or payments were made for authorship.

## CONFLICTS OF INTEREST

AM Zeidan: Leukemia and Lymphoma Society Scholar in Clinical Research. Research funding (institutional) from Celgene/BMS, AbbVie, Astex, Pfizer, Medimmune/AstraZeneca, Boehringer‐Ingelheim, Cardiff oncology, Incyte, Takeda, Novartis, Aprea, and ADC Therapeutics. Participated in advisory boards and/or had a consultancy with and received honoraria from AbbVie, Otsuka, Pfizer, Celgene/BMS, Jazz, Incyte, Agios, Boehringer‐Ingelheim, Novartis, Acceleron, Astellas, Daiichi Sankyo, Cardinal Health, Taiho, Seattle Genetics, BeyondSpring, Cardiff Oncology, Takeda, Ionis, Amgen, Janssen, Epizyme, Syndax, Gilead, Kura, Chiesi, ALX Oncology, BioCryst, Notable, Orum, and Tyme. Served on clinical trial committees for Novartis, AbbVie, Gilead, BioCryst, AbbVie, ALX Oncology, Geron, and Celgene/BMS. U Borate: Advisory board for Genentech, Daiichi Sankyo, Takeda, Pfizer, Novartis, Research funding from Novartis, Pfizer, Jazz, Kura Oncology, and Servier. Investigator in AbbVie‐funded Clinical Trials. DA Pollyea: Advisory board member for AbbVie, Jazz, Bristol Myers Squibb, Novartis, BeiGene, BerGenBio, Arcellx, Syros, Immunogen, Astra Zeneca, Notable Labs, Kura, Ryvu; Consultant for Syros, Genentech; Research Funding from Teva, Bristol Myers Squibb, Karyopharm and AbbVie. AM Brunner: Research funding to my institution from Novartis, Celgene, Takeda, AstraZeneca, and consulting for BMS/Celgene, Forty‐Seven, Inc, Gilead, Novartis, Takeda, Keros Therapeutics, Taiho, Agios, AbbVie, and Jazz Pharmaceuticals. F Roncolato: Investigator in AbbVie‐funded Clinical Trials. Jacqueline S Garcia: Institutional research funding (for trials) from AbbVie, Genentech, Pfizer, Prelude, Astra Zeneca; advisory board for AbbVie, Astellas, Takeda. R Filshie: Investigator in AbbVie‐funded Clinical Trials. T Odenike: Consulting or Advisory Role: AbbVie, Celgene; Research Funding: Celgene (Inst), Incyte (Inst), Astra Zeneca, Astex Pharmaceuticals (Inst), NS Pharma (Inst), AbbVie (Inst), Gilead Sciences (Inst), Janssen Oncology (Inst), Oncotherapy (Inst), Agios (Inst), CTI/Baxalta (inst). A‐M Watson: Received Travel Support from Roche, Amgen. R Krishnadasan: Investigator in AbbVie‐funded Clinical Trials. A Bajel: Honoraria—AbbVie, Amgen, Astellas Novartis, Pfizer, Takeda Speaker Fees—Amgen. K Naqvi: Employee of Genentech and may own Roche stock or options. J Zha, WH Cheng, Y Zhou, D Hoffman, JG Harb, J Potluri: Employees of AbbVie and may own stock. G Garcia‐Manero: Research support from Genentech and AbbVie.

## Supporting information


**FIGURE S1** Study design
**FIGURE S2.** Marrow complete remission rates with venetoclax and azacitidine treatment. (A) Response rates. (B) Duration of response
**FIGURE S3.** Clinical activity in mutational subgroups and risk categories
**FIGURE S4.** Survival in patients by baseline IPSS‐R and blast count percent
**TABLE S1.** Baseline and clinical characteristics of venetoclax monotherapy
**TABLE S2.** Summary of treatment‐emergent adverse events (TEAEs) leading to study drug discontinuation, interruption, dose reduction, and death
**TABLE S3.** Efficacy of patients treated with venetoclax monotherapy
**TABLE S4.** Patient responses by baseline mutational status in patients treated with venetoclax and azacitidine
**TABLE S5.** Venetoclax pharmacokinetic parameters on cycle 2 day 4

## Data Availability

AbbVie is committed to responsible data sharing regarding the clinical trials we sponsor. This includes access to anonymized, individual, and trial‐level data (analysis data sets), as well as other information (e.g., protocols, clinical study reports, or analysis plans), as long as the trials are not part of an ongoing or planned regulatory submission. This includes requests for clinical trial data for unlicensed products and indications. This clinical trial data can be requested by any qualified researchers who engage in rigorous, independent, scientific research and will be provided following review and approval of a research proposal, Statistical Analysis Plan (SAP), and execution of a Data Sharing Agreement (DSA). Data requests can be submitted at any time after approval in the US and Europe and after acceptance of this manuscript for publication. The data will be accessible for 12 months, with possible extensions considered. For more information on the process or to submit a request, visit the following link: https://www.abbvieclinicaltrials.com/hcp/data‐sharing/.
