# Supplementary material for: A phase 1b study of venetoclax and azacitidine combination in patients with relapsed or refractory myelodysplastic syndromes
Source: Am J Hematol. 2022 Nov 10;98(2):272–81. doi: 10.1002/ajh.26771 (PMC10100228; doi:10.1002/ajh.26771)
Supplement: Supplementary file 1 — FIGURE S1 Study design FIGURE S2. Marrow complete remission rates with venetoclax and azacitidine treatment. (A) Response rates. (B) Duration of response FIGURE S3. Clinical activity in mutational subgroups and risk categories FIGURE S4. Survival in patients by baseline IPSS‐R and blast count percent TABLE S1. Baseline and clinical characteristics of venetoclax monotherapy TABLE S2. Summary of treatment‐emergent adverse events (TEAEs) leading to study drug discontinuation, interruption, dose reduction, and death TABLE S3. Efficacy of patients treated with venetoclax monotherapy TABLE S4. Patient responses by baseline mutational status in patients treated with venetoclax and azacitidine TABLE S5. Venetoclax pharmacokinetic parameters on cycle 2 day 4 [file AJH-98-272-s001.docx]

**SUPPLEMENTAL APPENDIX**

**A phase 1b study of venetoclax and azacitidine in patients with relapsed or refractory myelodysplastic syndromes**

Contents

[Supplemental Figure 1. Study design 2](#_Toc116547890)

[Criteria for dose-limiting toxicities 2](#_Toc116547891)

[Recommendations for Dose Adjustment Due to Haematological Toxicity 3](#_Toc116547892)

[Supplemental Table 1. Baseline and clinical characteristics of venetoclax monotherapy 4](#_Toc116547893)

[Supplemental Table 2. Summary of treatment-emergent adverse events (TEAEs) leading to study drug discontinuation, interruption, dose reduction, and death 6](#_Toc116547894)

[Supplemental Table 3. Efficacy of patients treated with venetoclax monotherapy 7](#_Toc116547895)

[Supplemental Table 4. Patient responses by baseline mutational status in patients treated with venetoclax and azacitidine 9](#_Toc116547896)

[Supplemental Figure 2. Marrow complete remission rates with venetoclax and azacitidine treatment A. Response rates B. Duration of response 10](#_Toc116547897)

[Supplemental Figure 3. Clinical activity in mutational subgroups and risk categories 11](#_Toc116547898)

[Supplemental Figure 4. Survival in patients by baseline IPSS-R and blast count percent 12](#_Toc116547899)

[Supplemental Table 5. Venetoclax pharmacokinetic parameters on cycle 2 day 4 13](#_Toc116547900)

# Supplemental Figure 1. Study design


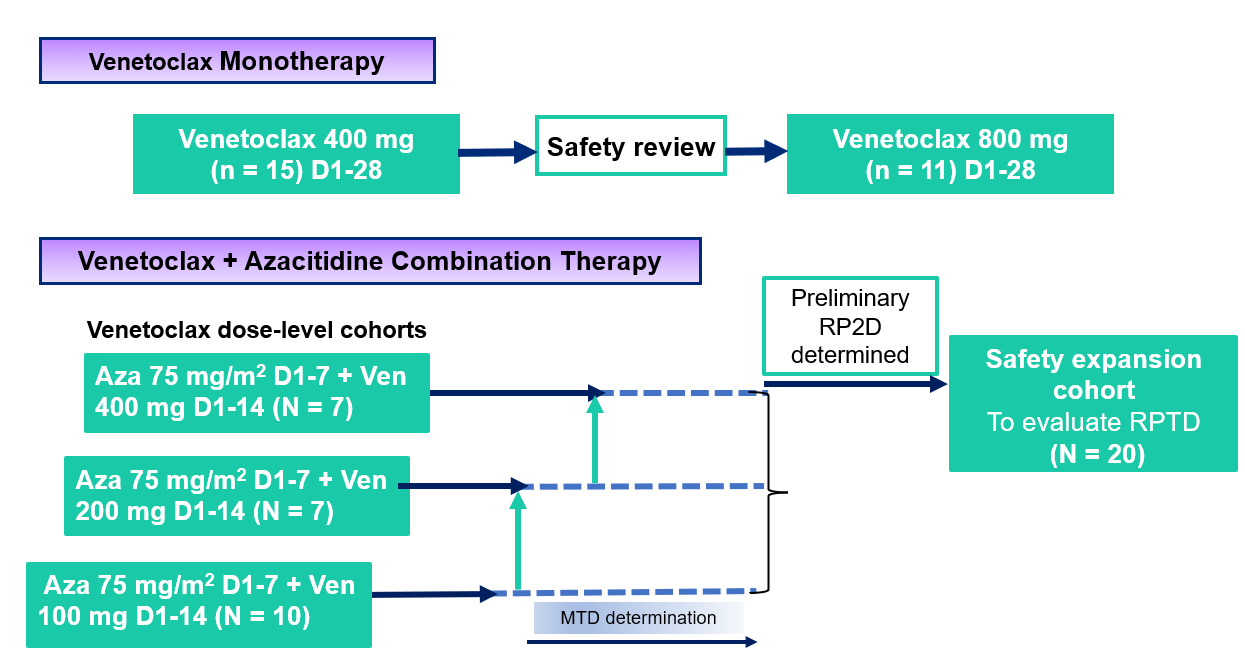


# Criteria for dose-limiting toxicities

Dose limiting toxicities (DLTs) for dose-escalation purposes were determined based on adverse events that occurred during the DLT observation period. For this study, the DLT observation period was defined as the first treatment cycle. Adverse events occurring after the DLT observation period were also reviewed and taken into consideration for dose-escalation decisions.

Any of the following events were considered a DLT unless the investigator attributed the event to a clearly identifiable cause such as underlying illness or disease progression, concurrent other illness, or concomitant medication:

● Grade ≥ 3 neutropenia (Absolute neutrophil count [ANC] < 1 × 10^9/L), which did not recover as defined below

- Recovery was defined as an increase in ANC from nadir by ≥ 50% of the reduction from baseline to nadir or any increase to an ANC of ≥ 1 × 10^9/L within 42 days from Day 1 of Cycle 1
- Delayed recovery was deemed a DLT if it was due to treatment-induced toxicity, as confirmed by a bone marrow sample showing an overall bone marrow cellularity of < 5%

● Grade ≥ 3 thrombocytopenia associated with clinically significant bleeding that required transfusion of platelets over routine or baseline transfusion needs based on the investigator's opinion

● Grade ≥ 4 non-haematologic toxicity deemed related to venetoclax per investigator's opinion

Grade 4 infections or complications related to infections (e.g., sepsis) were deemed DLTs if it was due to treatment-induced neutropenia, as confirmed by a bone marrow sample showing an overall bone marrow cellularity of < 5%.

# Recommendations for Dose Adjustment Due to Haematological Toxicity

For a treatment-emergent decrease of the ANC during venetoclax dosing (Days 1 – 14 of

a given cycle) to < 0·1 × 10^9/L or a platelet count of < 5 × 10^9/L interruption of

venetoclax was recommended if the bone marrow sample indicated treatment-induced

toxicity. For patients who experienced an adverse event that resulted in delay of the subsequent

treatment cycle, the dosing duration of venetoclax could be reduced from 14 to 9 days for

the next cycle per investigator’ discretion. For patients experiencing a DLT,

venetoclax could be restarted at a reduced dose level. For subsequent cycles with improved

recovery, re-escalation of venetoclax dose and dosing duration to 14 days could be

considered. Any DLT occurring during the administration period of venetoclax (the first 14 days of a

given cycle unless shortened) required interruption of venetoclax. If the DLT occurred during the period of combined administration of azacitidine and venetoclax (the first 7 to 9 days of a given cycle), interruption of azacitidine could also be necessary. A delay in treatment to allow for recovery from haematological toxicity affected both agents, azacitidine and venetoclax. Dose modifications of azacitidine after

Cycle 1 was implemented according to the local most recent azacitidine prescribing information and per investigator’s discretion.

# Supplemental Table 1. Baseline and clinical characteristics of venetoclax monotherapy

| **Characteristic** | **Venetoclax monotherapy**  **(N = 26)** |
| --- | --- |
| **Sex, n (%)** |  |
| Male | 21 (80·8) |
| Female | 5 (19·2) |
| **Race** |  |
| White | 24 (92·3) |
| Black or African American | 1 (3·8) |
| Asian | 1 (3·8) |
| **Age, years, median (range)** | 77 (58 ─ 88) |
| **ECOG performance score, n (%)** |  |
| 0 | 2 (7·7) |
| 1 | 22 (84·6) |
| 2 | 2 (7·7) |
| **Bone marrow blasts, n (%)** |  |
| < 5% | 12 (46·2) |
| ≥ 5% to 20% | 14 (53·8) |
| **Median bone marrow blast count, % (range)** | 5·0 (0·0 ─ 19·0) |
| **Number of prior therapies, median (range)** | 1 (1 ─ 4) |
| **Number of prior HMA therapies, n (%)** |  |
| 1 | 25 (96·2) |
| 2 | 1 (3·8) |
| **HMA failure, n (%)** |  |
| Primary | 19 (73·1) |
| Secondary | 0 |
| Other^a^ | 7 (26·9) |
| **Type of prior HMA therapy, n (%)** |  |
| Azacitidine | 18 (69∙2) |
| Decitabine | 6 (23∙1) |
| **Number of cycles of prior HMA therapies, n (%)** |  |
| ≤ 6 | 9 (34·6) |
| > 6 | 17 (65·4) |
| **IPSS cytogenetic risk categories, n (%)** |  |
| Good | 13 (50·0) |
| Intermediate | 5 (19·2) |
| Poor | 8 (30·8) |
| **IPSS-R risk categories, n (%)** |  |
| Low | 3 (11·5) |
| Intermediate | 2 (7·7) |
| High | 6 (23·1) |
| Very high | 10 (38·5) |
| Missing/Not evaluable | 5 (19·2) |
| **Genetic mutations^b^, n (%)** |  |
| *TP53* | 10 (38·5) |
| *RUNX1* | 0 |
| *TET2* | 6 (23·1) |
| *ASXL1* | 9 (34·6) |
| *SRSF2* | 2 (7·7) |
| *IDH2* | 1 (3·8) |
| *SF3B1* | 3 (11·5) |
| *DNMT3A* | 0 |
| *EZH2* | 2 (7·7) |
| *BCORL1* | 2 (7·7) |
| Not detected/missing/not evaluable | 1 (3·8) |

ECOG, Eastern Cooperative Oncology Group; HMA, hypomethylating agent; IPSS-R, International Prognostic Scoring System – Revised; mCR, complete remission with incomplete marrow remission; RBC, red blood cell

^a^Other: Either primary or secondary HMA failure; **^b^** Mutations assessed from bone marrow and peripheral blasts

# Supplemental Table 2. Summary of treatment-emergent adverse events (TEAEs) leading to study drug discontinuation, interruption, dose reduction, and death

|  | **Venetoclax 100 mg + Azacitidine 75 mg/m^2^ (N = 10) n (%)** | **Venetoclax 200 mg + Azacitidine 75 mg/m^2^ (N = 7) n (%)** | **Venetoclax 400 mg + Azacitidine 75 mg/m^2^ (N = 27) n (%)** | **All Venetoclax 400 mg + Azacitidine 75 mg/m^2^  (N = 44) n (%)** | **Venetoclax monotherapy (400 mg or 800 mg)**  **(N = 26) n (%)** | **All patients (N = 70) n (%)** |
| --- | --- | --- | --- | --- | --- | --- |
| Any TEAE leading to venetoclax discontinuation | 2 (20·0) | 0 | 7 (25·9) | 9 (20·5) | 4 (15·4) | 13 (18·6) |
| Any TEAE leading to azacitidine discontinuation | 1 (10·0) | 0 | 6 (22·2) | 7 (15·9) | 0 | 7 (10·0) |
| Any TEAE leading to venetoclax interruption | 6 (60·0) | 3 (42·9) | 12 (44·4) | 21 (47·7) | 10 (38·5) | 31 (44·3) |
| Any TEAE leading to azacitidine interruption | 6 (60·0) | 2 (28·6) | 10 (37·0) | 18 (40·9) | 0 | 18 (25·7) |
| Any TEAE leading to venetoclax dose reduction | 0 | 0 | 0 | 0 | 0 | 0 |
| Any TEAE leading to azacitidine dose reduction | 0 | 0 | 4 (14·8) | 4 (9·1) | 0 | 4 (5·7) |
| Any TEAE leading to venetoclax dose duration  reduction | 1 (10·0) | 1 (14·3) | 3 (11·1) | 5 (11·4) | 0 | 5 (7·1) |
| Any TEAE leading to death | 1 (10·0) | 0 | 3 (11·1) | 4 (9·1) | 2 (7·7) | 6 (8·6) |
| Deaths from any cause | 6 (60·0) | 5 (71·4) | 18 (66·7) | 29 (65·9) | 19 (73·1) | 48 (68·6) |

# Supplemental Table 3. Efficacy of patients treated with venetoclax monotherapy

|  | **Venetoclax monotherapy**  **(N = 26)** |
| --- | --- |
| **Response rates, n (%)** |  |
| Modified overall response rate (CR + PR+ mCR) | 1 (3·8) |
| Complete remission (CR) | 0 |
| Marrow complete remission (mCR) | 1 (3·8) |
| **Median time to first response for mCR, months (95% CI)** | 1·6 (1·6 ─ 1·6) |
| **Median duration of response for CR + mCR, months (95% CI)** | 3·9 (NE ─ NE) |
| **Composite response of mCR + HI**^a^**, n (%)** |  |
| mCR + HI^b^ (HI-E + HI-P + HI-N) | 0/1 |
| CR + PR + mCR + HI^b^, n/N (%) | 3 (11·5) |
| **Post-baseline transfusion independence (TI)**^c^**rate, n/N (%)** |  |
| RBC | 1 (3·8) |
| Platelet | 2 (7·7) |
| RBC and platelet | 9 (34·6) |
| **Maximal duration of post-baseline TI, months, median (95% CI)** |  |
| RBC | 3·4 (2·1 ─ 4·6) |
| Platelet | 4·6 (2·0 ─ 9·2) |
| RBC and platelet | 2·8 (2·8 ─ 2·8) |
| **Post-baseline TI**^c^ **rate for patients who were transfusion dependent at baseline, n/N (%)** |  |
| RBC transfusion dependent at baseline | 1/24 (4·2) |
| Platelet transfusion dependent at baseline | 3/14 (21·4) |
| RBC or platelet transfusion dependent at baseline | 1/24 (4·2) |
| **Maximum duration of post-baseline TI for patients who were transfusion dependent at baseline, months (range)** |  |
| RBC | 4·6 (4·6 ─ 4·6) |
| Platelet | 2·1 (2·0 ─ 5·3) |
| RBC and platelet | 2·8 (2·8 ─ 2·8) |
| **Transformation from MDS to AML, n (%)** | 5 (19·2) |
| **Time to AML transformation, months, median (range)** | 1·6 (0·7 ─ 6·5) |
| **Time to next treatment, months, median (95% CI)** | 4·2 (2·3 ─ 6·1) |
| **Median overall survival, months, median (95% CI)** | 6·7 (3·8 ─ 19·7) |
| **Median progression-free survival, months, median (95% CI)** | 3·4 (1·6 ─ 5·6) |
| **Median event-free survival, months, median (95% CI)** | 3·4 (1·6 ─ 5·5) |

CR, complete remission; mCR, marrow complete remission; NE, not evaluable; HI, haematological improvement; PR, partial remission

^a^Patients who achieved any component of (CR + mCR + PR + HI)

^b^HI = HI-E + HI-P + HI-N; HI-E, Transfusion dependent on packed red blood cells or whole blood 8 weeks prior to cycle 1 day 1 (C1D1) or haemoglobin level < 11 g/dL; HI-P, Transfusion dependent on platelet 8 weeks prior to C1D1 or platelet counts < 100 X 10^9/L; HI-N, Neutrophil counts < 1.0 X 10^9/L at baseline

^c^The post-baseline transfusion independence rate is defined as a period of at least 56 days with no transfusion during the evaluation period. The evaluation period for transfusion independence is from the date of the first dose of the study drug to the last dose of the study drug + 30 days or one day before the date of progressive disease from disease response (2006) eCRF, death or the initiation of post-treatment therapy whichever is earliest.

# Supplemental Table 4. Patient responses by baseline mutational status in patients treated with venetoclax and azacitidine

| **Mutations^a^** | **CR + mCR**  **rate, n (%)** | **Duration of response (CR/mCR), median, months (95% CI)** | **Progression-free survival,**  **median, months (95% CI)** | **Overall survival,**  **median, months (95% CI)** |
| --- | --- | --- | --- | --- |
| *TP53* (n = 5) | 1 (20) | 6·02 (NE ─ NE) | 5·07 (5·03 ─ NE) | 5·92 (5·05 ─ NE) |
| *TET2* (n = 6) | 2 (33) | 8·55 (NE ─ NE) | 8·26 (3·55 ─ NE) | 12·27 (8·26 ─ NE) |
| *ASXL1* (n = 17) | 7 (41) | 6·81 (6·22 ─ NE) | 6·94 (4·57 ─ NE) | 12·63 (9·21 ─ NE) |
| *DNMT3A* (n = 7) | 4 (57) | 9·52 (6·32 ─ NE) | 6·48 (5·07 ─ NE) | 13·42 (5·92 ─ NE) |
| *IDH2* (n = 6) | 5 (83) | 13·01 (6·22 ─ NE) | 14·11 (13·42 ─ NE) | 17·07 (13·42 ─ NE) |
| *STAG2* (n = 14) | 7 (50) | 13·19 (8·55 ─ NE) | 9·24 (6·94 ─ NE) | 17·42 (12·24 ─ NE) |
| *SRSF2* (n = 6) | 3 (50) | 7·24 (7·24 ─ NE) | 8·82 (4·18 ─ NE) | NR (11·28 ─ NE) |
| *RUNX1* (n = 11) | 6 (54) | 6·22 (4·74 ─ NE) | 6·94 (5·43 ─ NE) | NR (14·34 ─ NE) |
| **%BCL-2+/%BCL-_X_L+ Blasts** |  |  |  |  |
| %BCL-2+/%BCL-_X_L+ Blasts > Median  (n = 16) | 8 (50) | 9·52 (6·02 ─ NE) | 7·17 (5·66 ─NE) | 14·11 (6·91 ─ NE) |
| %BCL-2+/%BCL-_X_L+ Blasts < Median  (n = 14) | 5 (36) | 10·92 (6·38 ─ NE) | 9·24 (3·55 ─ NE) | 14·1 (12·24 ─ NE) |

CR, complete remission; mCR, marrow CR; NE, not evaluable; NR, not reached

**^a^** Mutations assessed from bone marrow and peripheral blasts

# Supplemental Figure 2. Marrow complete remission rates with venetoclax and azacitidine treatment A. Response rates B. Duration of response


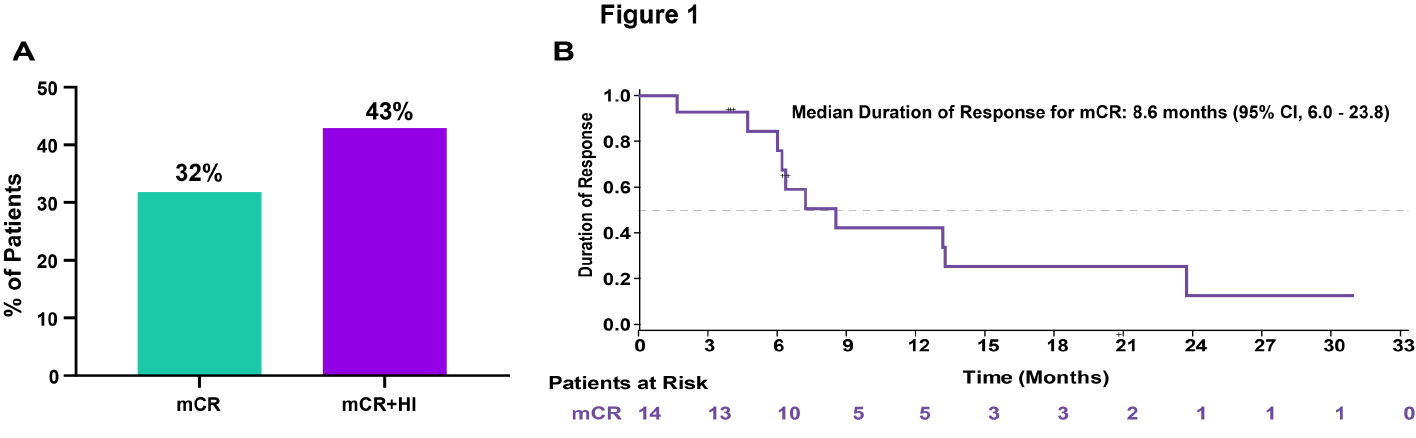


CR, complete remission; mCR, marrow complete remission; HI, haematological improvement

# Supplemental Figure 3. Clinical activity in mutational subgroups and risk categories


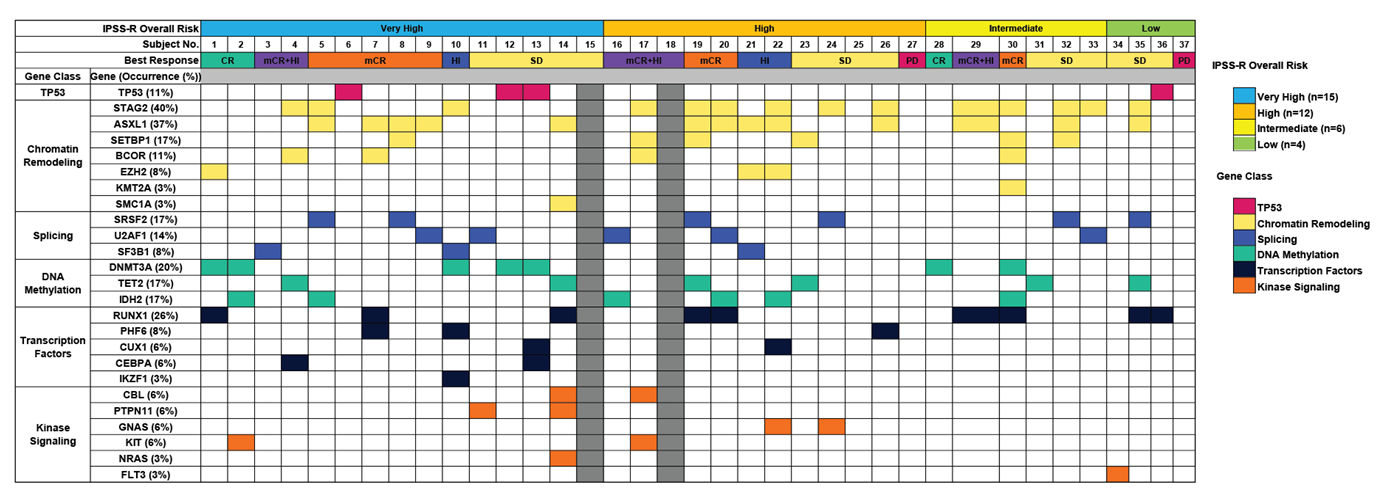


Note: Heat map describes baseline mutation status for 35 of 37 patients who were evaluable for a clinical response

# Supplemental Figure 4. Survival in patients by baseline IPSS-R and blast count percent


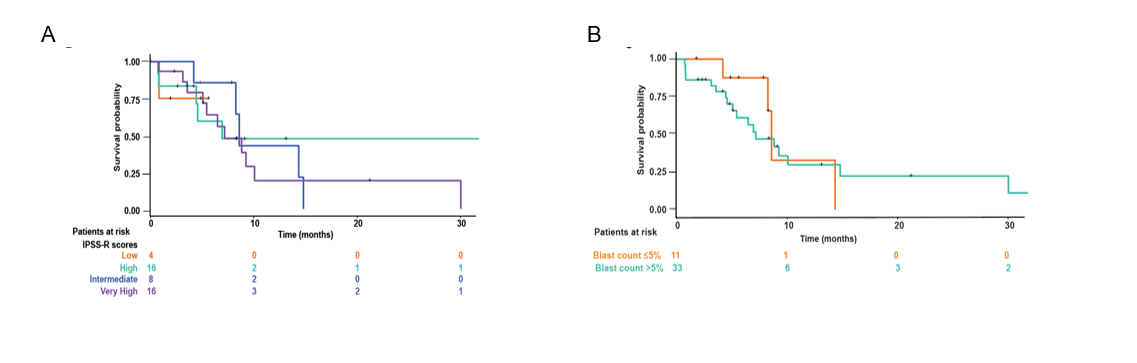


1. Median overall survival by baseline IPSS-R risk categories; B. Median overall survival by baseline blast count %

# Supplemental Table 5. Venetoclax pharmacokinetic parameters on cycle 2 day 4

| **Pharmacokinetic Parameter (Unit)** | **Venetoclax monotherapy (N = 17)** | **Venetoclax+Azacitidine  (N = 14)** |
| --- | --- | --- |
| T_max_ (h) | 6·0 (4·0 ─ 8·0) | 6·0 (0·0 ─ 24·0) |
| Dose-normalised C_max_ (ng/mL/mg) | 4·85 (43) [1·77 ─ 9·40] | 4·17 (40) [1·75 ─ 7·26] |
| Dose-Normalised AUC_24_ (ng•h/mL/mg) | 77·1 (44) [32·0 ─ 149]^a^ | 63·3 (39) [32·8 ─ 104]^b^ |

Median (range) for T_max_, geometric mean (CV%) [range] for dose-normalised C_max_ and AUC_24_.
T_max_ = time to maximum observed plasma concentration; C_max_ = maximum observed plasma concentration; AUC_24_ = area under the plasma concentration-time curve from time 0 to 24 hours

^a^ n = 16; ^b^ n = 13
